# Characterization of bacterial community shift in human Ulcerative Colitis patients revealed by Illumina based 16S rRNA gene amplicon sequencing

**DOI:** 10.1186/1757-4749-6-22

**Published:** 2014-06-14

**Authors:** Sandeep A Walujkar, Dhiraj P Dhotre, Nachiket P Marathe, Parimal S Lawate, Renu S Bharadwaj, Yogesh S Shouche

**Affiliations:** 1 National Centre for Cell Science, Sai Trinity Complex, Sutarwadi Road, Pashan Gaon, Pashan, 411021 Pune, Maharashtra, India; 2 Department of Medical Microbiology, B.J. Govt Medical College, Pune, Maharashtra, India; 3 Department of Gastroenterology, Dr. ParimalLawate Gastroenterology Clinic, Pune, Maharashtra, India

**Keywords:** Inflammatory disease, Bacterial community shift, 16S rRNA gene, High through-put sequencing, QIIME analysis

## Abstract

**Background:**

The healthy human intestine is represented by the presence of bacterial communities predominantly belonging to obligate anaerobes; however disparity and dysanaerobiosis in intestinal microflora may lead to the progression of ulcerative colitis (UC). The foremost aim of this study is to consider and compare the gut microbiota composition in patients suffering from different stages of UC.

**Methods:**

This study represents data from the biopsy samples of six individuals suffering from UC. The samples were collected by colonoscopy and were processed immediately for isolation of DNA. Mucosal microbiota was analyzed by means of 16S rRNA gene-based Illumina high throughput sequencing. Quantitative real-time PCR (qPCR) was performed to determine total bacterial abundances.

**Results:**

Analysis of 23,927 OTUs demonstrated a significant reduction of bacterial diversity consistently from phylum to species level (p < 0.05) for individuals suffering from severe stage of UC. Significant increase in abundance of unusual aerobes and facultative anaerobes, including members from the phylum Proteobacteria (p- = 0.031) was also observed. A 10 fold increase in the total bacterial count was detected in patients suffering from severe inflammatory stage (2.98 +/-0.49 E + 09/ml) when compared with patients with moderate (1.03+/-0.29 E + 08/ml) and mild (1.76 +/-0.34 E + 08/ml) stages of inflammation.

**Conclusion:**

The reduction of bacterial diversity with an increase in the total bacterial count indicates a shift of bacterial communities which signifies dysbiosis and dysanaerobiosis at the mucosal level for patients suffering from UC.

## Introduction

Prevalence of Functional Gastrointestinal Disorders (FGID) in western countries is very high, with Inflammatory Bowel Disease (IBD) being prevalent throughout North America and Europe [1]. Crohn’s Disease (CD) and Ulcerative Colitis (UC) are two separate chronic entities of IBD; both have common features but can be differentiated due to the respective nature of inflammation and specific disease locations [1-3]. During active CD, any part of the gastrointestinal tract (i.e., from mouth to anus) may get affected while UC is restricted to the colon and the rectum. When categorized by microscopic features, UC is restricted only up to the mucosal or epithelial lining of the gastrointestinal tract, while CD may affect the whole bowel wall and can cause serious transmural lesions [1,4-7].

IBD is postulated to be associated with industrialized nations, with very less data being available from developing countries [1]. Although recent studies have shed light on the role of commensal bacteria intrinsic to the gastrointestinal tract in the pathogenesis and etiology of IBD [1,7-13] the peculiar nature of dysbiosis that occurs in the microbiota of gastrointestinal tract during IBD remains to be expounded [4].

The gut of an infant is sterile at the time of birth [14-16]. The initial step of colonization in infants involves colonization by facultative anaerobes such as *Escherichia coli* or *Enterococci*. Gradually, with the increased number of these facultative anaerobes and as available oxygen is consumed, a favorable environment is created for subsequent colonization by obligate anaerobes such as *Bifidobacteria*, *Bacteroides*, and *Clostridia*[8]*.* By the age of four, the human gut microbiota becomes fully mature. From this age, every individual develops a unique and complex gut microbiota which remains stable throughout adulthood [2,14,16-19]. These complex microbial communities have evolved and developed persistently in shaping up the mucosal immune system during the early phase of life. Absence of these intestinal microbial communities leads to defective cell mediated immune response, discontinuous cytokine production, reduction of total mucosal cell turnover and muscle wall thickness, thereby, giving rise to various autoimmune diseases [3,8,9,16,20,21].

Some of the recent studies have also indicated the crucial role of phyla Proteobacteria in the pathogenesis of UC [22]. Proteobacteria is the largest and most diverse bacterial phyla with known clinical importance in human gastrointestinal diseases, and are implicated in luminal dysbiosis leading to the imbalance between the plausible pathogenic bacteria and functionally defensive commensal bacteria [22-24].

From the experiments performed so far on animal models of IBD, it is apparent that very few signs of inflammation are observed in germ-free animals as compared to the animals that harbour natural microflora [4,8,11]. Many comparative studies of gut microbiota of patients with IBD and non-IBD controls have been directed towards determination of specific core microbiota or assigning tentatively a particular group, genus, species or strain of microorganism to the prognosis of IBD [8,9,13,25]. These studies have clearly marked the imbalance or dysbiosis in the gut microbiota of patients suffering from either CD or UC [8-11,25]. In addition, one of the contemporary study has also proved that the microbiota composition in healthy and diseased individuals is influenced by ethnic and geographical factors [26], thus it becomes more pertinent to study the microbiota composition from different geographical and ethnic niches.

Collectively, all these studies confirm the changes which occur in the gut microbial communities in UC patients as compared to healthy controls. [8-11,25] However, these cross-sectional studies in which the disease status is neglected can lead towards complicated outcome, very few studies, have considered the role of mucosal microbiota in relation with the severity of disease [27]. Studies which investigate the compositional microbiota with changes in disease status are currently inadequate.

Therefore, the principal aim of the current study is to evaluate and compare the differences between the mucosa associated microbiota of patients manifesting mild, moderate, and severe stage of UC, as defined by a Simple Clinical Colitis Activity Index (SCCAI) ≥ 5 and Baron Score for UC [4,12,28-30]. We adapted two independent techniques to assess and correlate specific bacterial groups in colonic mucosal biopsy samples (collected in a manner that precisely maintained the composition of the microbiota). Amplicon libraries of 16S rRNA genes were generated by Illumina-based deep sequencing method, which were subsequently used to demonstrate the differences in taxonomic diversity of microbial communities in patients suffering from the three different stages of UC. We also applied quantitative real-time polymerase chain reaction (qPCR) to quantify the total bacterial abundance among selected sub-sets of samples. The present findings demonstrate data from Indian patients with significant irregularities in the intestinal microbiota for the first time. Our study shows a reduction in the overall diversity of the microbial community with increasing disease severity. We also find a concurrent decrease of dominant obligate anaerobes and an increase in the population of unusual aerobes and other facultative anaerobes. This typical abnormal condition may be termed as dysanaerobiosis and could have a role in exacerbation of UC, and other colonic diseases.

## Results

### Subject characteristics

The study population represents equal number of males and females, and their disease status was categorised based upon the respective SCCAI and Baron scores. Other demographics characters and body mass index (BMI) were similar in the three study groups (Table 1). Intestinal mucosal biopsy samples were initially collected from eleven individuals within a period of sixteen months, but the final number of samples for the study were reduced to six based on our stringent inclusion criteria (details in material & methods section).

**Table 1:** Essential characteristic of patients and biopsy tissue at time of sampling

**No.**	**Diagnosis**	**Age**	**Sex**	**Biopsy site**	**Inflammation**	**Disease stage**	**SCCAI Score**	**Baron score**
SP1	UC	42	M	Sigmoid	Acute	Initial	≥ 2	1
SP2	UC	34	F	Rectum	Acute	Initial	≥ 2	1
SP3	UC	28	M	Sigmoid	Acute	Moderate	≥ 3	2
SP4	UC	41	F	Rectum	Acute	Moderate	≥ 3	2
SP5	UC	47	M	Descending Colon	Chronic	Severe	≥ 5	3
SP6	UC	40	F	Descending Colon	Chronic	Severe	≥ 5	3

UC: Ulcerative Colitis; SCCAI: Simple Clinical Colitis Activity Index scores for ulcerative colitis ≥ 5.

### Operational taxonomic unit (OTU) analysis: abundance and prevalence

**Figure 1:**
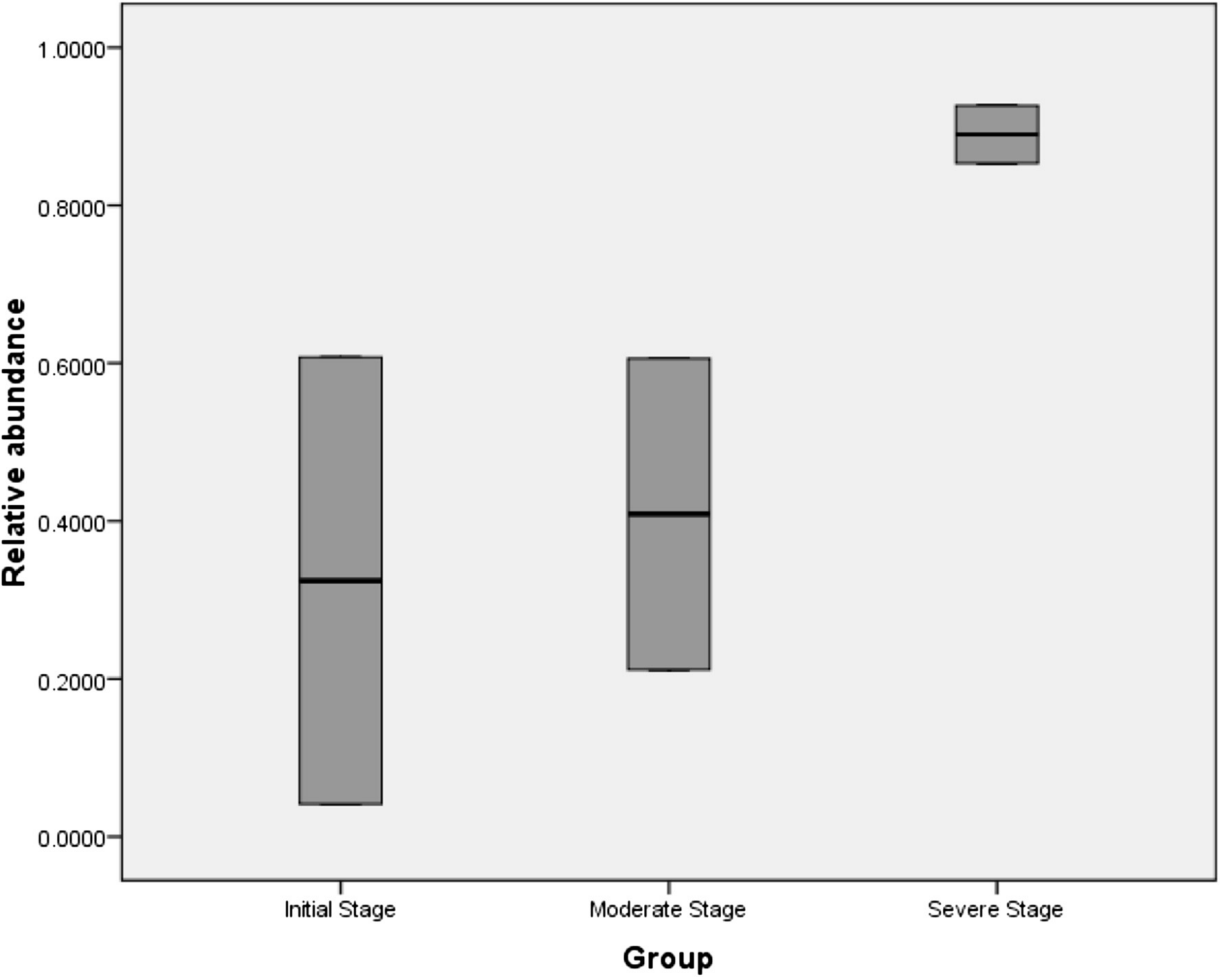
**Box Plot showing the overall prevalence of phylum Proteobacteria in samples from UC patients at three different clinical stages (*p*-value = 0.031).**

**Figure 2:**
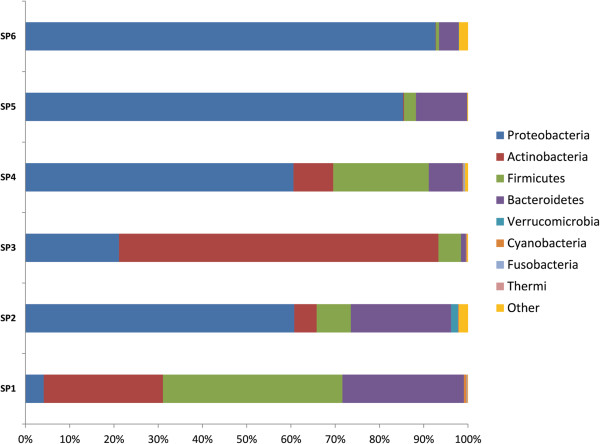
**Relative abundance of OTUs showing the distribution at taxonomic level of bacterial phyla in UC patients at three different disease stages.** Samples – SP5 & SP6 – Severe stage of UC, SP4 & SP3 – Moderate stage of UC, SP2 & SP1- Mild stage of UC.

**Figure 3:**
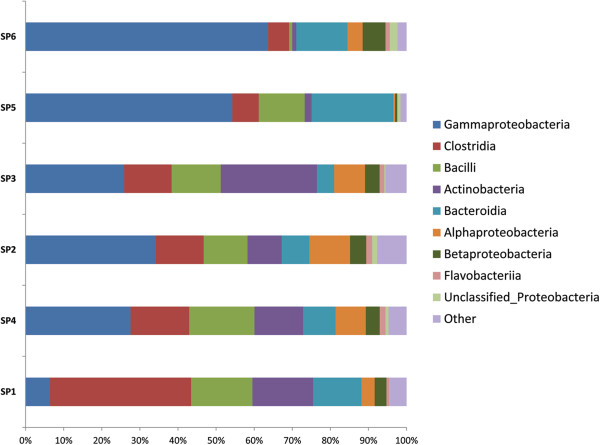
Class-level distribution of OTUs which displays the abundance of Class Gammaproteobacteria over other bacterial classes in patients suffering from severe stage (SP5 & SP6) of UC as compared to Moderate (SP3 & SP4) and mild disease stage (SP1 & SP2) of UC.

For obtaining a pattern of total richness of the microbial communities based on operational taxonomic unit (OTU) analysis, DNA from the biopsy samples of six individuals was used as described above (or in M&M). A total of 2,271,930 good quality sequence reads were generated. Clustering of all reads with a 97% pairwise-identity cut-off resulted in a total of 23,927 OTUs. All reads with less than 97% similarity with the known organisms in Greengenes database were not considered for further analysis. Representative sequences for each OTU were assigned to different bacterial taxonomic levels by using Greengenes database release (May 2013). Twenty two different bacterial phyla were identified. The most abundant phylotypes found in patients with severe stage of UC (sample SP5 and SP6) belonged to Proteobacteria at phylum level (Figure 1), followed by Bacteroidetes, while OTUs representing Firmicutes and other phyla were negligible. In patients categorized with moderate or intermediate stage UC (sample SP3 and SP4), no clear trend was observed in the distribution of OTUs, however, the abundance of OTUs was again observed to be higher within the phylum Proteobacteria. The abundance of phylotypes belonging to Actinobacteria also increased considerably, while significant reduction of phylotypes affiliated to Bacteroidetes and Firmicutes was observed. Similarly, samples from the patients that were characterized with mild stage UC (sample SP1 and SP2) showed abundance of Bacteroidetes and Firmicutes to be higher than or equal to that of Proteobacteria and Actinobacteria (Figures 2 and 3).

### Analysis of the mucosa-associated intestinal microbiota

The diversity of mucosa-associated microbiota from the descending colon, sigmoid colon and rectum biopsy specimens of patients suffering from three different stages of UC were compared. Despite a definite degree of inter-group variation in the microbiota between patients, the distribution of OTUs showed a significant association with the severity of disease. A steady decrease in the proportion of Firmicutes, and a sharp increase in Proteobacteria (p = 0.031) especially the Gammaproteobacteria (p = 0.042) was clearly observed between mild and severe stage biopsy samples of UC. The percentage of Firmicutes was notably high in the UC patients manifesting a mild stage compared to that of a severe stage. Moreover, a shift in the mucosa associated microbiota from obligately anaerobic bacterial community to facultative anaerobes, and a simultaneous increase in the unusual aerobic bacterial community members is clearly evident among patients at two different inflammatory stages (Additional file 1: Table S1).

Of the 162 different types of bacterial families observed during our analysis, 67 families represented more than 1% for any reference OTU in any given sample and were considered for further analysis. These 67 bacterial families were then broadly categorized as belonging to either obligate anaerobes, facultative anaerobes or aerobic group of bacteria. According to our analysis, 26 bacterial families had significant *P values < 0.05* (refer Additional file 1: Table S1), while the remaining 41 families, including *Bacteroidaceae, Clostridiaceae, Prevotellaceae, Ruminococcaceae, Peptostreptococcaceae, Bacillaceae, Enterobacteriaceae, Pasteurellaceae, Alcaligenaceae, Pseudomonadaceae* and *Xanthomonadaceae* were represented by higher number of OTUs, but had *P values > 0.05. This difference could arise due to the* variation in the bacterial diversity observed between individuals within the same inflammatory stage. The abundance of OTU’s representing the families *Enterobacteriaceae, Pasteurellaceae, Alcaligenaceae, Pseudomonadaceae,* and *Xanthomonadaceae* was observed to be at higher levels in the individuals suffering from chronic inflammatory stage as compared to the acute inflammatory stage during UC. (Additional file 2: Table S2).

### PCoA analysis

**Figure 4:**
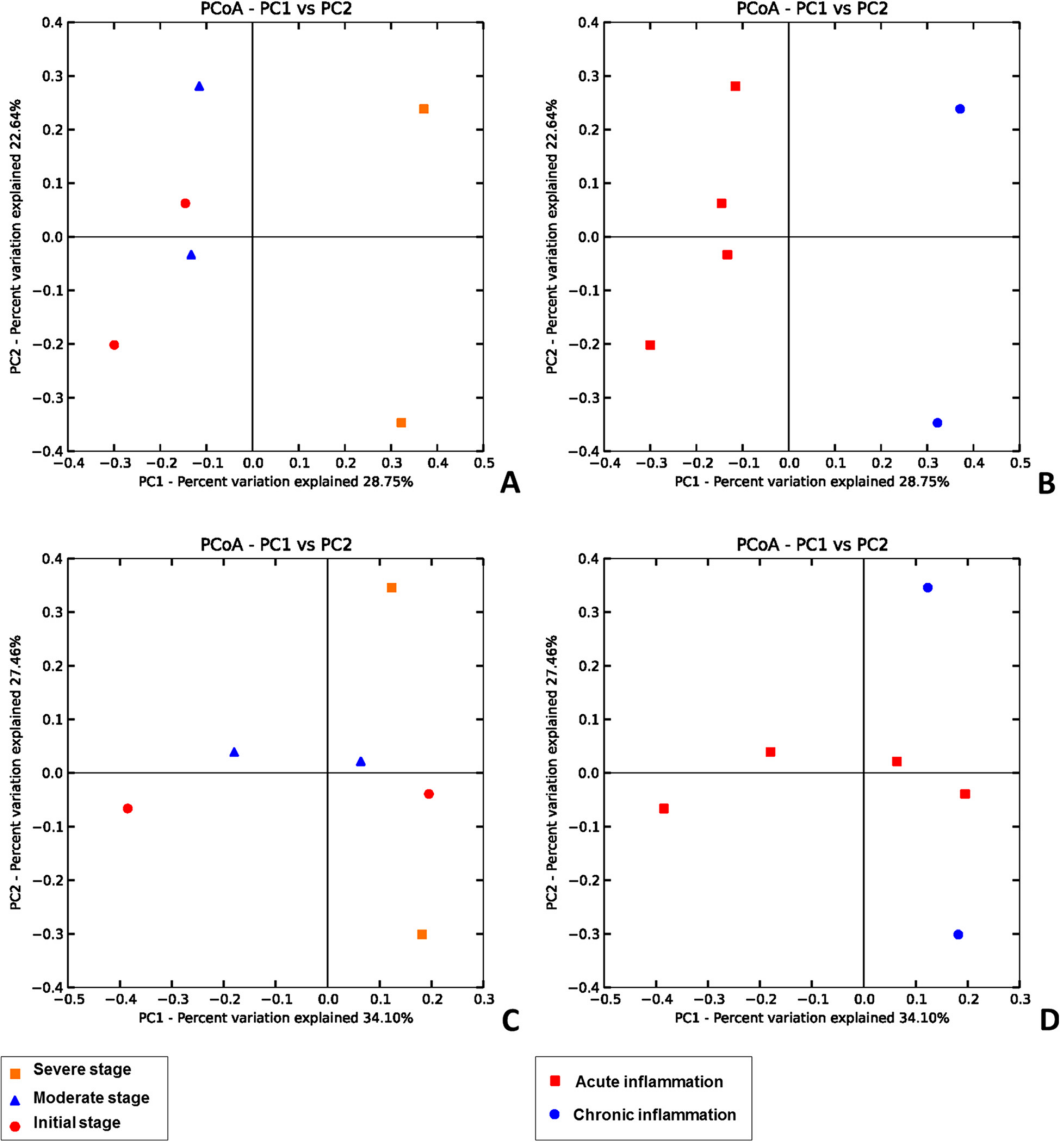
**Principal Coordinate Analysis (PCoA)of samples calculated with QIIME. (A & C)** PCoA Plots based on unweightedUniFrac metrics for all the samples.PCoA plots explain the total bacterial diversity observed in each sample. **(B & D)**PCoA Plots based on weighted UniFrac metrics for all the samples. PCoA plots explain the total bacterial abundance observed in each sample.

Two dimensional Principal Coordinates Analysis (PCoA) plots of weighted and unweightedUniFrac distances were used to visualize complex relationships in the microbial communities between three different clinical stages of UC (Figure 4). According to Figure 4A& C individuals suffering from severe stage of UC show different pattern of clustering as compared to individuals suffering from moderate and mild stage of UC, while in Figure 4B & D no specific clustering pattern is observed for relative abundance of OTUs for different disease stages. Based on Weighted Unifrac Figures 4B & D it can be speculated that even though the relative abundance of OTUs in patients suffering from severe stage of UC are not similar with each other but are quite different as compared to initial and moderate stages of UC (Figure 4).

### Quantification of bacterial population

**Figure 5:**
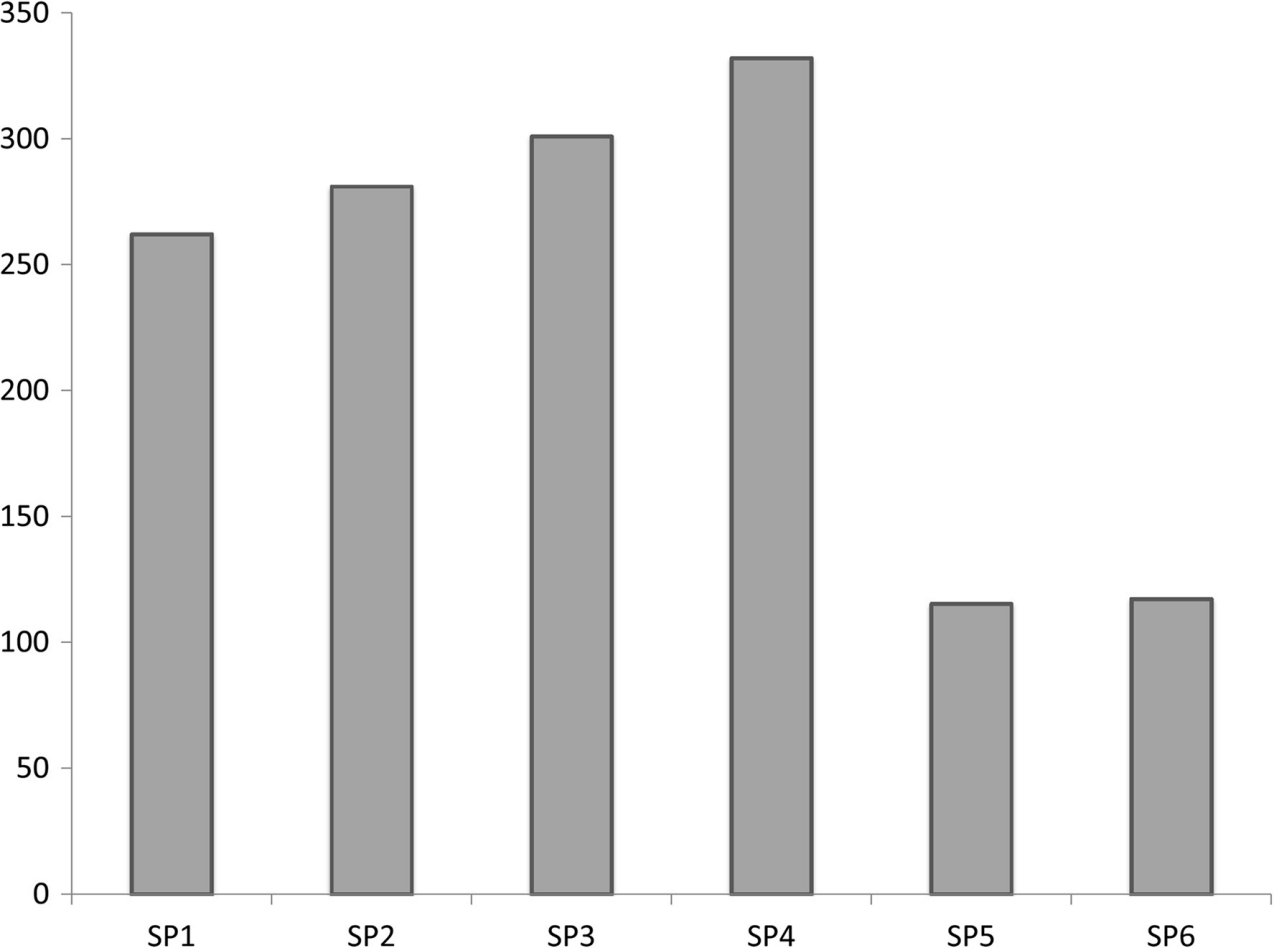
Total species level bacterial richness as observed in patients suffering from mild (SP1 & SP2), moderate (SP3 & SP4) and severe (SP5 & SP6) stages of ulcerative colitis

**Figure 6:**
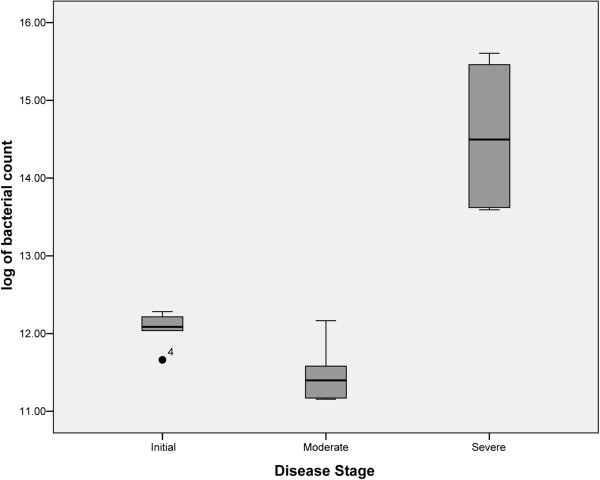
Box Plot showing variation between total bacterial loads in samples from patients at mild (SP1 & SP2), moderate (SP3 & SP4) and severe (SP5 & SP6) stages of ulcerative colitis.

Quantitative PCR (qPCR) analysis of total bacterial count was performed for all the UC sub-set samples. Our results showed that the intestinal biopsies of the patients suffering from severe UC contained more number of bacteria, which may be from less diverse bacterial communities (Figure 5), whearas the bacterial load for samples with moderate and mild stage of UC were 10 fold lower compared to severe stages (Figure 6).

## Discussion

Many of the previous studies have compared patients suffering from UC and healthy controls, and have positively established that the gut microbiota plays a crucial role in the maintenance of health, and is vital to disorders such as UC [11,15,16,31]. These studies have also identified the fact that in healthy controls, the integral complex community of intestinal microbiota is predominantly constituted by members of phyla Firmicutes and Bacteroidetes, while Actinobacteria, Proteobacteria, Fusobacteria, Verrucomicrobia, and Cyanobacteria have also been detected in humans but only in smaller proportions [8,9,24,32,33].

Other studies have shown differences in microbiota by comparing the data generated from the patients suffering with active UC and inactive UC. For example, the loss of bacterial community belonging to clostridial cluster XIVa has been associated with development of active UC [34]. The role of *F. parusnitzii* have been implicated in the exacerbation of UC [35] while another study have shown reduction in Firmicutes/Bacteroidetes ratio which was found to be conjugated with the active UC disease state [36]. All these previous reports have considered UC patients without differentiating them on the basis of disease severity, which make them susceptible towards selection bias. Only one recent report has shed some light on the compositional shift and decreased diversity of some of the bacterial communities compared with the changing disease status [27]. Here, Andrea K. Bartram et al., have grouped the patients with the changing disease course and have used fecal microbiota to study the changes in the bacterial diversity in patients suffering from IBD within the groups, without using any healthy controls [27]. Another study have investigated the colonic microbiota of a single patient with UC (12 year old girl), and have only compared their findings with previously available references [37]. In the current study, we have used biopsy samples (mucosal microbiota) of UC patients and classified them on the basis of disease severity, which may give an insight about the shift in bacterial community. This study enlightens the dysbiosis occurring between the mucosal microbiota of patients suffering from UC with increase in the severity of the disease.Although limited by the sample size, to our knowledge this is the first high-throughput sequencing study that gives an in-depth view of tentative mucosal associated microbiota involved with the prognosis of UC in Indian patients. Our sequencing results suggest an episode of reduction in bacterial diversity from phylum to species level. Only one hundred species were detected among the individuals suffering from a severe stage of UC as compared to approximately two to three hundred species that are observed in individuals manifesting a mild or moderate stage of UC. This disparity in mucosa associated bacterial diversity is clearly indicated by OTU based two dimensional PCoA plots (Figure 4). Considering mild stages of UC as disease control, further analysis indicates a gradual reduction in the phyla Bacteroidetes and Firmicutes, and an increase in the members of phyla Proteobacteria with increase in severity of the disease. Although the bacterial diversity in all the six patients may have differed, there is certainly a dysbiosis, and a particular group of bacterial community (especially Proteobacteria) starts dominating as the disesase progresses. In the patients suffering from mild and moderate stage of UC, the unstability of bacterial diversity may be due to the competition of the different bacterial communities to dominate/establish in a particular niche i.e. the human intestine (Figures 2 and 3). Results from qPCR based study of intestinal biopsies samples demonstrate that the bacterial count in patients suffering from severe stage of UC is much higher (tenfold) when compared to that of patients categorized under mild or moderate stage of UC, which may be from less diversified bacterial communities, i.e. bacteria belonging to phyla Proteobacteria would have outnumbered the other bacterial communities normally prevalent in mucosa associated gastrointestinal microbiota (Figure 6).

Similar shifts in composition have been reported by other investigators using both culture-dependent and molecular techniques [5,13,26,38,39]. However, none of the previous studies could confirm a specific bacterial species or a core group of bacterial community to be associated with the etiology of IBD.

Results obtained from the current study indicate that a particular species may not be solely responsible in pathogenesis of UC they point towards the imbalance or dysbiosis which is observed in gut microbiota, involving the depletion of obligate anaerobes and an unusual increase in facultative anaerobes and a few aerobic species (Additional file 3: Table S3). This profound disparity of the gastrointestinal tract may be responsible in activation of various reactive oxygen species and a subsequent increased oxygen tension in the gastrointestinal environment. Therefore, the disparity of the gastrointestinal tract may be considered an important component in the prognosis of UC.

## Conclusions

Our data demonstrates a dysbiosis and dysanaerobiosis in the bacterial community profile of patients suffering from mild, moderate, and severe stages of Ulcerative Colitis. The sequencing study signifies the decline in the bacterial community belonging to the phyla Firmicutes and Bacteroidetes and an unusual increase in Proteobacteria among diseased mucosal intestinal niches, thereby, suggesting a decrease in the influence of obligate anaerobes and an increasing influence of facultative anaerobes and some aerobic bacterial communities. The clinical relevance of this study still needs to be addressed as it is difficult to establish the complex relation between host and microbes and whether the state of dysanaerobiosis in the gastrointestinal tract causes exacerbation of UC. Further in-depth investigations comparing mucosal-associated intestinal microbiota with additional number of patients with UC can give better insights.

## Material and methods

### Subject characteristics

All the eleven patients included in this study were selected from those undergoing colonoscopy from Dr. Parimal Lawate’s Gastroenterology Clinic, Pune, Maharashtra, India. A written informed consent was obtained from each patient, and the study was granted ethical approval by the B.J Govt Medical and Sasoon General Hospitals Ethical Committee (Ref No. BJMC/IEC/Pharmac/D0311013-13). Patient details including age, sex and the site of the colon from where the biopsies were taken are indicated in Table 1. All the procedures were carried out after preparing the colon with two bottles (60 ml) of EXELYTE (Oral buffered saline) solution mixed with 300 ml of sterile flavored water on the day of colonoscopy. Patients were kept on liquid diet (Water, Coconut water, Lemon juice) to avoid excess dehydration. Two colonic mucosal biopsy samples from the same mucosal area of approximately 1 × 2 mm size were collected from each subject. Each biopsy was collected in 1 ml of sterile phosphate buffer saline solution, and biopsies were then weighed and almost immediately processed for DNA extraction. The extracted DNA was preserved at -20°C until further experiments were performed.

Special care was taken for selection of patients, and only those individuals who had not received antibiotics in the past 90 days prior to sample collection were selected. Three out of eleven patients had not provided exact information about intake of any sort of antibiotic or steroid as medicine and thereby were excluded from the study. Inclusion criteria comprised of subjects with an approximate age group between 25 to 45 years of age with any gender but with the same ethnicity. All individuals were subjected to a clinical investigation by a gastrointestinal physician to exclude the diagnosis of Crohn’s disease, inflammatory bowel syndrome, celiac disease, food nutrient malabsorptions, or any other intestinal abnormality. Two patients were detected to be suffering from multiple bowel disorder, thus these two patients were also excluded from the study. The remaining six UC patients had active GI symptoms at the time of sample collection as confirmed by standard clinical, endoscopic, radiological and histopathological criteria.

A gastrointestinal pathologist assigned scores to all the biopsy samples for the presence of ulceration, and acute or chronic inflammation. Simple Clinical Colitis Activity Index score (SCCAI) and modified Baron Score with a severity scale ranging from 0–5 was assigned to each sample, where a score of 5 represented the most severe form of UC. Scores obtained from both the above mentioned scaling methods were used for gradation of the severity value for each biopsy used in the study [12,28,29,40,41].

### DNA extraction and Illumina library generation

Total DNA was extracted from each biopsy sample using QIAamp Tissue DNA extraction Kit (Qiagen) according to the manufacturer’s instructions. Purified DNA samples were subjected to agarose gel electrophoresis and Nanodrop (Thermo Scientific) analysis for integrity check and qualitative verification respectively. For each sample, 250 ng/μl of DNA was extracted and subjected to PCR on a 9700 thermo cycler (Applied biosystems). The V3 region of the 16S rRNA gene was amplified using 341 F and 534R primers [42].

Four PCR amplifications were carried out for each sample, using 50 μl reaction mixtures. Each reaction mixture contained 25 pmol of each primer, a 200 μM concentration of each deoxynucleoside triphosphate (dNTP), 1.5 mM MgCl2, and 1 U.

Phusion Hot Start II High-Fidelity DNA polymerase (Thermo Scientific). The optimum PCR conditions comprised of an initial denaturation step at 95°C for 5 min followed by 20 cycles of 95°C for 1 min, 50°C for 1 min, and 72°C for 1 min and ended with an extension step at 72°C for 7 min. The products were then separated from the primer-dimers by electrophoresis on a 2% agarose gel. PCR products of the appropriate size were recovered using a QIAquick gel extraction kit (Qiagen, Mississauga, Ontario, Canada). For each library, quadruplet PCR products for each biopsy sample with unique identification were mixed in equal nanograms quantities. The sequencing was carried out at Xcelris Genomics Labs, Ahmedabad, India. The library was clonally amplified on a cluster generation platform using Illumina, version 4, and cluster generation reagents to attain a target density of approximately 150,000 clusters per tile in a single channel of a flow cell. The resulting library was then sequenced on Illumina Hi-Seq 2000 (platform), sequencing reagents, generating paired reads of 125 bases. After sequencing was complete, image analysis, base calling, and error estimation were performed using Illumina Analysis Pipeline (version 2.6) [43-45]. All sequences were submitted to DDBJ Sequence Read Archive with accession number as [DDBJ: DRA001221].

### Initial quality filtering

Using a custom algorithm PANDAseq [46], paired end Illumina reads were assembled according to index sequence. If a mismatch was observed, the paired-end sequences involved in the assembly were discarded. All sequences with ambiguous base calls were also discarded.

### Bioinformatics analysis

Sequences were assigned to operational taxonomic units (OTUs) by using a closed reference-based OTU picking method in QIIME v1.7. Greengenes database available on May, 2013 was used for OTU picking [43,44]. Sequences from the study were assigned to a reference sequence using the UCLUST [47] with 97% similarity threshold. Sequences that did not have 97% identity to any of the reference sequences in the Greengenes database were not assigned to OTUs and thus, not considered further in these analyses. Classification of sequences was performed in MOTHUR v1.25. UnweightedUniFrac analysis was performed on tables of OTU counts. UniFrac performs a pairwise comparison of all communities in a data set, defining the overall degree of phylogenetic similarity between any two communities based on the degree of branch length they share on a bacterial tree of life [43,45,48]. Since sampling depth can impact UniFrac values, and thus clustering patterns [43,48] data was normalized to the 197,067 reads (i.e. the least number of reads in sample SP3) before performing the PCoA analyses. All analyses were carried out using QIIME v1.7. [44] Taxa summaries at the all taxonomic level were performed using the RDP classifier trained on the May, 2013 Greengenes 97% reference data set using QIIME v1.7.

### Quantitative real-time PCR

Total bacterial quantification for all the six biopsies samples categorised according to severity index, into three disease stage’s i.e. severe, moderate, and mild stage of UC, was performed by Quantitative PCR (qPCR). Thermo cycler 7300 (Applied Biosystems, Foster City, CA) was used to perform all the qPCR, in conjunction with sequence detection system (SDS) version 1.4 qPCR Software. Each PCR was carried out in a final volume of 20 μl and contained the following: 1 × SYBR green qPCR Master Mix (Qiagen), 0.5 μM of each primer and 40 ng of purified colonic mucosal DNA. The thermal cycling conditions were 50°C for 2 minutes and 95°C for 5 minutes followed by 40 cycles of denaturing at 95°C for 15 seconds, primer annealing at 60°C for 30 seconds and DNA extension at 72°C for 90 seconds. Each plate included triplicate reactions per DNA sample and the appropriate set of standards. A 466-bp fragment of the bacterial 16S rRNA gene was amplified using the forward primer 5′ TCCTACGGGAGGCAGCAGT-3′ and the reverse primer 5′ -GGACTACCAGGGTATCTAATCCTGTT-3′ [49]. Extracted DNA from a pure *Bacteroidesfragilis* (CCUG 4856) culture was prepared into a series of tenfold dilution in RNase-free water and was used as a positive control in order to make a standard curve. For each reaction, a threshold of luminescence was determined and compared to the standard curve. Melting curve analysis of the PCR products was conducted following each assay to confirm that the fluorescence signal originated from specific PCR products and not from primer-dimers or other artefacts. All qPCR plates included a ‘no template’ negative control for each primer set. The abundances of each bacterial group in UC patients were expressed as a ‘fold change’ with respect to the other group.

### Statistical analysis

Statistical analysis was performed in R statistical package [50]. Good’s coverage, chao index, simpson’s index, simpson’s reciprocal index, student’s *T*-test and ANOVA analyses were performed to check if the differences observed at the family level were significant or not (*p*-value < 0.05). Alpha- and beta-diversity analyses were performed in QIIME v1.7.

## Competing interests

The authors declare that they have no competing interests.

## Authors’ contributions

SW, NM, YS and RB conceived and designed the study. PL provided the samples. PL and SW were involved in selection of patients and samples for the study. SW carried out the experiments and collected data. SW, DD and RB analysed and interpreted the data. SW performed the literature survey. SW and DD generated figures and tables. SW wrote the manuscript with inputs from RB, YS and DD. YS, SW and RB had full access to all of the data in the study and take responsibility for the integrity of the data and accuracy of the data analysis. All authors gave their final approval of the submitted and published versions.

## Supplementary Material

Additional file 1: Table S1 Distribution of 26 families *(P< 0.05)* with average reference OTUs between two inflammatory stages. Click here for file

Additional file 2: Table S2 Average OTU Abundance observed at family level between two inflammatory stages. Click here for file

Additional file 3: Table S3 Total bacterial count by Real time PCR. Click here for file
