# Supplementary material for: Characterization of bacterial community shift in human Ulcerative Colitis patients revealed by Illumina based 16S rRNA gene amplicon sequencing
Source: Gut Pathog. 2014 Jun 14;6:22. doi: 10.1186/1757-4749-6-22 (PMC4094692; doi:10.1186/1757-4749-6-22)
Supplement: Additional file 3: Table S3 — Total bacterial count by Real time PCR. [file 1757-4749-6-22-S3.docx]

**Table S3**: Total bacterial count by Real time PCR.

| **SampleID** | **Disease Stage** | **Average copy number µl/ml** | **Standard Error** |
| --- | --- | --- | --- |
| **SP1** | Initial/Mild | 1.76E+05 | 3.34E+04 |
| **SP2** |  |  |  |
| **SP3** | Moderate | 1.03E+05 | 2.69E+04 |
| **SP4** |  |  |  |
| **SP5** | Severe | 2.98E+06 | 4.91E+05 |
| **SP6** |  |  |  |
